# The Genetic Transformation Mechanism and Application of Plant Hairy Roots

**DOI:** 10.3390/plants15142160

**Published:** 2026-07-13

**Authors:** Haolin Liu, Zhengyang Zhang, Guangya Bian, Ping Li

**Affiliations:** 1 College of Forestry, Hebei Agricultural University, Baoding 071000, China; liuhaolin202511@163.com; 2 Key Laboratory of National Forestry and Grassland Administration on Colorful Tree, Baoding 071000, China; 3 Hebei Key Laboratory of Floral Biological Breeding, Baoding 071000, China; 4 National Engineering Research Center for Floriculture, School of Landscape Architecture, Beijing Forestry University, Beijing 100083, China; zhangzhengyang2025@163.com; 5 Shijiazhuang Academy of Agriculture and Forestry Sciences, Shijiazhuang 050899, China

**Keywords:** plant genetic transformation, *Rhizobium rhizogenes*, plant hairy root, genetic breeding, secondary metabolites

## Abstract

Innovation in plant-breeding technology relies on precise, targeted genetic manipulation, which depends on the efficient delivery of genetic elements and robust plant cell regeneration systems. *Agrobacterium tumefaciens*, a traditional medium for genetic material delivery, is well established and has become widely adopted, but it faces limitations such as species specificity, transformation chimerism, and the requirement for two-step processes of transformation and regeneration. In contrast, *Rhizobium rhizogenes* can deliver genetic material with efficiency comparable to that of *A. tumefaciens* while excelling in producing transgenic homozygotes in a single step. This article summarizes the molecular mechanisms underlying *R. rhizogenes*-mediated hairy-root induction in plants, discusses the development of genetic material delivery systems, details the construction of hairy-root induction and regeneration platforms, explores molecular mechanisms in hairy roots and their applications in genetic breeding, and highlights the use of hairy roots for extracting secondary metabolites. The hairy-root genetic transformation system provides efficient, simplified, and broadly adaptable tools for genetic manipulation in breeding, accelerating the advancement of cutting-edge technologies such as gene editing, rapid domestication, and synthetic biology-driven breeding.

## 1. Introduction

With the continuous development of breeding technology, modern plant breeding has entered the era of ‘precise design’. Breakthroughs in gene-editing techniques such as CRISPR/Cas9 have provided technical support for the directional improvement of plant traits [1]. However, construction of an efficient and stable plant genetic transformation system is a prerequisite for accurate modification of plant traits. The plant genetic transformation system is an important technological advancement in modern science [2,3]. It serves as an essential agricultural biotechnology for crop improvement, enabling quality enhancement, yield optimization, secondary-metabolite enrichment in medicinal plants, and sustainable agricultural development [4]. The main plant genetic transformation approaches include Agrobacterium-mediated, gene gun bombardment, and nanoparticle delivery methods. However, Agrobacterium-mediated transformation remains the predominant approach in plant transgenics due to its technical maturity, low equipment requirements, and cost-effectiveness [5]. This method relies on *A. tumefaciens* or *R. rhizogenes* as a carrier to introduce foreign genes into plant cells and integrate them into the genome [6,7]. Agrobacterium-mediated transformation requires simultaneous optimization of the transformation scheme and an efficient plant regeneration system to be successful [8,9]. The plant regeneration system is time-consuming, species-limited, and prone to chimeras, issues that have become key factors restricting conversion efficiency. Conversely, *R. rhizogenes*-based transformation systems circumvent the requirement for plant regeneration by directly generating transgenic hairy-root cultures in host plants. This method can greatly simplify operations and shorten the experimental cycle [9].

Research on plant hairy-root genetic transformation systems can be traced back to the early 1990s, yet it initially attracted little attention in the scientific community [10]. The plant hairy-root genetic transformation system—now a novel, highly developed system—offers advantages such as high efficiency, operational simplicity, and genetic stability. As genetic-engineering technology has advanced, researchers have focused on efficiently introducing and stably expressing exogenous genes in plant cells. In this context, some researchers have conducted extensive studies on the mechanism of root induction in plants by the *R. rhizogenes* root-inducing (Ri) plasmid [11]. After years of exploration, the system has matured and become an important plant-genetic-transformation tool [12]. This technology has now been successfully applied to numerous species, including in Fabaceae, Asteraceae, Brassicaceae, Solanaceae, and woody plants [13,14,15].

This review systematically integrates, for the first time, the latest advances in three emerging fields—gene editing, secondary-metabolite production, and industrial dye bioremediation—using hairy roots and thoroughly analyzes the key bottlenecks hindering their commercialization, providing a comprehensive, forward-looking theoretical framework for interdisciplinary applications of hairy-root technology.

## 2. Mechanism of *R. rhizogenes*-Mediated Adventitious Hairy-Root Formation in Plants

### 2.1. Structure and Function of R. rhizogenes

**Figure 1:**
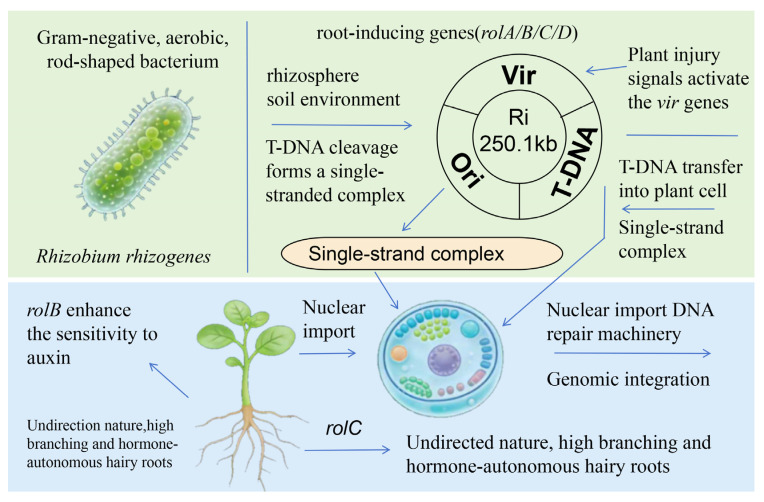
Molecular mechanisms of T-DNA transfer mediated by *R. rhizogenes* Ri plasmid and hairy-root regeneration. *R. rhizogenes* is a Gram-negative, aerobic, rod-shaped bacterium. This figure illustrates the process by which its Ri plasmid (≈250.1 kb) induces hairy-root formation in host plants via T-DNA integration upon infection. In the rhizosphere, plant wound signals activate the Vir region (vir operon) on the Ri plasmid, triggering cleavage of the T-DNA region—harboring root-inducing genes such as *rolA/B/C/D*—into single-stranded T-DNA complexes (T-strands). These complexes are subsequently translocated into plant cells through a type IV secretion system (T4SS), and following nuclear import, the integration of T-DNA into the host genome is mediated by the plant’s DNA double-strand-break repair machinery. For phenotypic induction, the integrated *rolB* gene enhances auxin sensitivity in plant cells, and in synergy with *rolC* and other *rol* genes, it ultimately drives the development of hairy roots with hallmarks of indeterminate growth, high branching, and auxin autonomy.

*R. rhizogenes* is a Gram-negative, aerobic, rod-shaped bacterium that occupies rhizospheric soil environments. *R. rhizogenes* belongs to the Rhizobiaceae family in terms of phylogeny and can specifically induce hairy-root formation by infecting plants [16]. The core functional element of this bacterium is a large covalently closed circular root-inducing (Ri) plasmid located outside the chromosome, with a size of 250.1 kb. Its structure is precisely divided into three key functional regions: the origin region (Ori), which is responsible for plasmid autonomous replication; the virulence (*vir*) gene region, encoding virulence proteins to mediate T-DNA processing and transmembrane transport; and the transfer DNA (T-DNA) region, containing root-inducing genes such as *rolA/B/C/D* [17,18]. When plants are damaged, they release phenolic compounds. These substances activate the *vir* genes on the Ri plasmid, causing the single-stranded T-DNA complex to be processed and transferred into the plant cells. This T-DNA complex then integrates into the plant genome through the DNA repair mechanisms of the host cell. Studies have shown that the integration of T-DNA mainly occurs through the non-homologous end-joining (NHEJ) pathway, which is a form of abnormal recombination that occurs at double-strand breaks (DSBs) in the host genome [19,20,21]. This process is mediated by host repair proteins (Ku70, Ku80, and DNA ligase IV) and does not require long sequence homology between the T-DNA and the integration site, a feature that distinguishes it from homologous recombination [22]. Recent studies have further demonstrated that PolQ (DNA polymerase θ) plays a crucial role in mediating the micro-homology-mediated end-joining (MMEJ) pathway, which may serve as an alternative integration pathway in certain genetic contexts [23,24]. The bacterial genome harbors virulence-determining genetic elements that orchestrate the metabolic reprogramming of infected host cells, redirecting plant biosynthetic pathways to facilitate the production of bacteria-specific metabolites. Hairy roots induced by *R. rhizogenes* infection in plants originate from a single plant cell, containing a set of chromosomes identical to that of the parent. They do not produce chimeras, ensuring genetic stability [25]. Moreover, hairy roots can grow normally and synthesize secondary metabolites vigorously on hormone-free culture media, making them suitable as a vector for the transfer of genetic material. Therefore, researchers began considering hairy roots not just as a neoplasm resulting from the attack of a pathogenic bacterium but also as a promising model for studying the features of secondary metabolism and, ultimately, as producers of natural products (Figure 1).

### 2.2. Mechanism of Hairy-Root Formation

*R. rhizogenes* can transform plant cells by transferring T-DNA from the Ri plasmid into the plant cell genome. The key lies in the presence of two functional regions associated with transformation on *R. rhizogenes*: the T-DNA and Vir regions [26]. During transformation, T-DNA enters plant cells and inserts itself into the host plant genome’s DNA region. The bacterium actively expresses a suite of pathogenicity genes responsible for inducing hairy-root proliferation and mediating opine biosynthesis in infected host tissues. The agropine Ri plasmid’s T-DNA has two independent regions, T_L_-DNA and T_R_-DNA, each with 25 bp repeat sequences at both ends [27]. These repeats are recognition sites used by restriction endonucleases to cleave T-DNA from the Ri plasmid. Each T-DNA fragment spans 15–20 kB and is separated by at least 15 kB of non-integrated plasmid DNA. Mutation analysis of TL-DNA identified four genetic loci—*rolA*, *rolB*, *rolC*, and *rolD*—affecting hairy-root induction. The TL-region’s complete nucleotide sequence shows 18 open-reading frames (ORFs), with ORFs 10, 11, 12, and 15 corresponding to the *rolA*, *rolB*, *rolC*, and *rolD* loci, respectively. *rolA*, *rolB*, and *rolC* are important for hairy-root induction [28]. In particular, *rolB* seems to be the most crucial in the differentiation of transformed cells, while *rolA* and *rolC* provide accessory functions. Although the T_R_-DNA is not essential for hairy-root formation, it has been shown that the *aux1* gene harbored in this segment provides the transformed cells with an additional source of auxin [29,30].

The Vir region assists the T-DNA region in completing the transformation process. The bacterial virulence system is encoded by a conserved seven-gene operon (*VirA-VirG*), whose gene products collectively mediate the precise excision and intercellular transfer of tumor-inducing (T-DNA) sequences [31]. The key pathway involved is the two-component signal transduction system, where *VirA* acts as the phenol receptor and *VirG* serves as the signal receiver [32]. In addition to these two genes, the other *Vir* genes play auxiliary but indispensable roles. When the bacterium attaches to the surfaces of plant cells, the injured plant cells become sensitive to *R. rhizogenes*, leading to the synthesis and release of phenolic compounds such as Acetosyringone [33]. At very low concentrations, acetosyringone acts as a chemoattractant and activates the *Vir* genes on the Ri plasmid, initiating the infection of T-DNA transfer. It binds to the expression product of *VirA*, activating other *Vir* genes, resulting in the cleavage, transfer, and eventual integration of T-DNA into the host cell genome. However, with a better understanding of the key factors or parameters required for successful infection of viable seeds and gene transfer, *R. rhizogenes* has also led to breakthroughs in monocot transformation [34].

**Figure 2:**
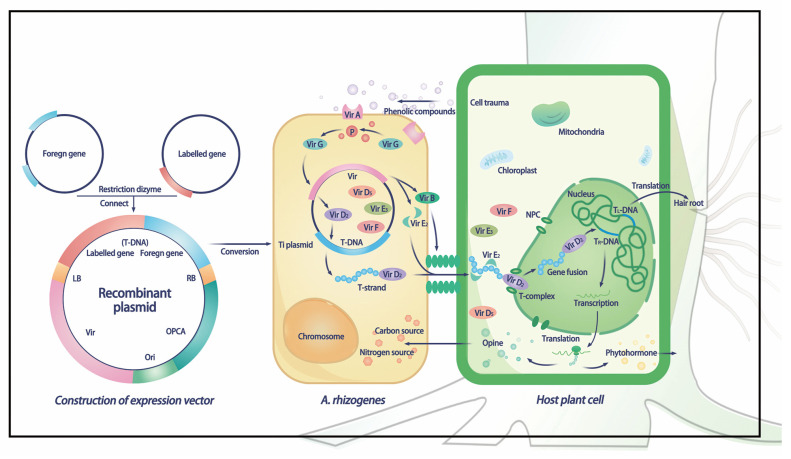
Schematic of the *R. rhizogenes*-mediated hairy-root transformation system and recombinant Ti expression vector construction. This workflow consists of three core stages: construction of the recombinant Ti plasmid expression vector, T-DNA transfer and delivery mediated by *R. rhizogenes*, and integration and expression of exogenous genes in host plant cells. First, the target foreign gene and labeled reporter gene are ligated into the T-DNA region between the left border (LB) and right border (RB) of the Ti plasmid via restriction endonuclease digestion and ligation to construct a recombinant expression vector containing a Vir region, replication origin (Ori), and opine synthesis-related gene (*OPCA*). After transformation into *R. rhizogenes*, phenolic compounds released from wounded plant cells bind to VirA receptor protein, activating VirG and triggering the expression of downstream Vir operons. The VirD1/VirD2 complex cleaves T-DNA to generate a single-stranded T-strand, which assembles with VirD2, VirE2, and other Vir proteins to form the T-complex. The T-complex is secreted into plant cytoplasm through the VirB/VirD4 type IV secretion system and then transported into the cell nucleus via nuclear pore complex (NPC). Subsequently, T-DNA carrying the foreign gene is integrated into the plant genome through gene fusion, followed by transcription and translation to produce phytohormones and opines. Accumulated phytohormones induce the formation of hairy roots, while opines serve as unique carbon and nitrogen sources for the growth of *R. rhizogenes*.

Consequently, during *R. rhizogenes*-mediated host infection, the root-inducing (Ri) plasmid is systematically translocated into plant cells through highly specialized molecular mechanisms. Subsequently, the *Vir* genes express a series of proteins that can break down the barriers of plant cell walls and membranes, allowing the plasmid to replicate inside the cell and integrate itself into the plant cell genome (Figure 2). This process is known as the gene transfer and integration (T-DNA) process. As the plasmid replicates inside the cell, the plant cell produces opines, which are signal molecules that induce cell dedifferentiation and the formation of hairy roots. Opines also serve as the sole carbon and nitrogen sources utilized by the *R. rhizogenes* bacteria. Under the influence of opines, the plant cells begin to dedifferentiate and form root-like structures known as hairy roots [35].

## 3. Factors Influencing Hairy-Root Induction

**Figure 3:**
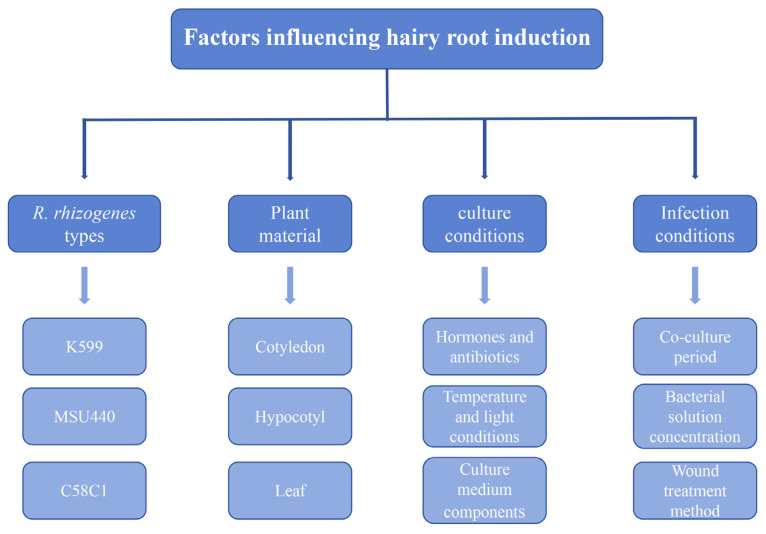
The main factors affecting the efficiency of hairy-root induction in plants.

The induction of hairy-root formation by *R. rhizogenes* is comprehensively regulated by multiple factors. The main influencing factors can be divided into four major categories: strain factors, plant material factors, infection conditions, and culture conditions (Figure 3) [36]. The genetic backgrounds of different strains and the variations in the Ri plasmids they harbor can significantly influence the expression patterns of *rol* genes, resulting in differences in transformation efficiency. The efficiency of hairy-root induction by *R. rhizogenes* varies across different plant species, a phenomenon that can be attributed to their distinct gene expression patterns, cellular physiological states, and stress response mechanisms. During infection, factors such as the concentration of the bacterial solution, the method of wound treatment, and the duration of co-culture all have a direct impact on the transfer efficiency of T-DNA, which, in turn, affects hairy-root induction efficiency. The composition of the culture medium, the content of hormones, the screening conditions for antibiotics, and the temperature and light conditions of the cultivation environment will also significantly affect the induction and subsequent growth of plant hairy roots.

### 3.1. R. rhizogenes Types

The efficiency of *R. rhizogenes* in inducing plant hairy roots is largely dependent on the type of Ri plasmid it carries. Different strains exhibit different infection capabilities and induction characteristics due to differences in plasmid structure and gene composition. The Ri plasmid is used to classify the *R. rhizogenes* strains into four main categories: the agropine (AR.QUAL, A4, A7, MSU440, C58C1, etc.), cucumopine (K599, etc.), mannopine (PRI8196, PRITR7, etc.), and heterocucumopine types (PRI1724, A13, etc.). The ability of different Agrobacterium strains to induce hairy-root formation in plants varies significantly [37]. According to phylogenetic analysis of the Ri plasmid, herterocucumopine and cucumopine Ri plasmids are grouped as type I, while mannopine and agropine are types II and III, respectively [38,39,40]. When an induction system for the hairy roots of *Liriodendron hybrid* was being established, significant differences in the transformation effects of different *R. rhizogenes* strains were found: K599 exhibited the highest induction ability and could the induce formation of a greater number of vigorous hairy roots. The induction efficiency of MSU440 was significantly lower, resulting in the formation of only a single root. The C58C1 system hardly produces any hairy roots that can be transformed [41]. When conducting the rapid transformation of peony using *R. rhizogenes*, researchers found that different strains of *R. rhizogenes* exhibited different transformation efficiencies: K599 showed a higher rate of inducing hairy-root formation and good growth conditions in rootstocks, seedlings, etc.; MSU440 and C58C1 had relatively lower or unstable induction efficiency; and A4 could induce formation of hairy roots, but this was often accompanied by unfavorable phenotypes such as excessive tissue proliferation [42]. Due to the K599 strain’s wide host range and high induction efficiency, it is widely used for inducing hairy roots.

### 3.2. Plant Material

The efficiency of inducing hairy-root formation is determined by multiple consecutive biological mechanisms. Whether *R. rhizogenes* can successfully infect plant cells is the starting point of the induction process. The differences in cell wall components, tissue structure, and phenolic secretions among various plant materials will directly affect the adhesion and invasion ability of *R. rhizogenes* [43,44,45]. Whether T-DNA can be successfully transferred and integrated is the molecular basis for the formation of hairy roots. Different plant species, genotypes, and types of tissue exhibit variations in aspects such as the strength of immune responses, the activity of DNA repair mechanisms, and the expression levels of nuclear localization proteins. These differences directly affect whether T-DNA can successfully enter and be integrated into the plant genome [20]. Whether plant cells can successfully differentiate into hair-like roots is the key to successful induction. Young tissues, due to their strong cell division ability and high endogenous auxin levels, are more likely to form root primordia, while mature or lignified tissues have weak regenerative capacity, and their induction rate is significantly reduced. An efficient hairy-root induction system was established in *Arabidopsis thaliana*. The young and mature roots of this species differ in terms of their cell wall structures and secretions, which directly affect the adhesion and infection efficiency of *R. rhizogenes* for root formation [46]. In one study, the hairy-root induction rates in different tissues of roses were compared. It was found that the leaf tissue, because of its thinner cell walls and abundant secretions, was more susceptible to infection, while the stem segments with a high degree of lignification had a lower induction efficiency [47]. There are significant differences in hairy-root induction efficiency among different genotypes of *Glycine max* [48], and there are considerable variations in transformation efficiency among *Gossypium hirsutum* varieties [49]. The leaf tissues of *Passiflora edulis* are more conducive to the integration of T-DNA and formation of hairy roots than the stem segments [50]. The influence of endogenous signals in *Zea mays* on T-DNA integration results in different transformation efficiencies for different genotypes of *Z. mays* [51].

### 3.3. Infection Conditions

In the process of genetic transformation mediated by *R. rhizogenes*, infection conditions are the key factors determining the success rate of hairy-root induction. A high concentration of bacterial suspension can lead to the death of explants, while a low concentration significantly reduces induction efficiency. Therefore, studies generally suggest that an optimal transformation effect can be achieved when the optical density (OD) is approximately 0.6 [52]. Additionally, the infection time also needs to be precisely controlled: too short a time can result in incomplete T-DNA transfer, while too long a time may cause cell damage and tissue necrosis [53]. Evidently, appropriate bacterial suspension concentrations and infection times not only affect the survival rate of explants but also directly determine the formation efficiency and stability of hairy roots. Therefore, systematic evaluation of infection conditions is an indispensable step in the optimization of different host plants and *R. rhizogenes* strains.

### 3.4. Culture Conditions

The culture environment also constitutes a critical factor influencing the transformation efficiency of *R. rhizogenes*. Studies have demonstrated that exposure to suboptimal conditions during explant culture frequently leads to overgrowth or induction failure, thereby compromising transformation success rates. Notably, traditional transformation systems for woody plants are often constrained by prolonged growth cycles and low regeneration efficiency. In contrast, optimized hairy-root systems enable acquisition of transgenic root systems within a shorter timeframe (approximately 2–3 months), offering a rapid platform for gene function analysis. Improvements in culture environments not only enhance explant survival rates and the stability of hairy-root formation but also, when integrated with gene-editing technologies, open new avenues for molecular design breeding in woody plants. Consequently, systematic optimization of culture conditions has emerged as a pivotal step in elevating hairy-root transformation efficiency and overcoming bottlenecks in the genetic improvement of woody plants.

Hormones play a crucial role in plant in vitro culture. Generally, the addition of different growth hormones can induce the formation of calluses, buds, and roots [54]. However, different plant tissues have different endogenous hormone levels, so the corresponding exogenous-hormone requirements in a medium vary [55]. For example, adding NAA benefits bud formation in *Cucurbita moschata* and *Citrullus lanatus* cotyledon nodes. The auxin-to-cytokinin ratio also affects explant regeneration. In *Coffea arabica*, a high-concentration auxin-induced callus is more conducive to later differentiation, while cytokinin weakens it [56]. Thus, the types and ratios of plant hormones added to the medium are key for explant induction and differentiation. The main exogenous hormones in plant tissue culture include auxins (like NAA, IAA, and IBA, which promote rooting) and cytokinins [57].

## 4. Genetic Transformation Systems of Plant Hairy Roots

The hairy-root genetic transformation system is widely employed in plant functional genomics and molecular breeding. Centered on the *R. rhizogenes*-mediated system, this approach features operational simplicity, high transformation efficiency, and robust stability of transgenic lines [58]. Compared with other transformation systems, it is more suitable for plant species with long transformation cycles and high technical complexity, and it has successfully been established as a relatively stable transformation system in hundreds of plant species (Table 1) [59].

### 4.1. Transformation Methods and Operation Procedures

**Figure 4:**
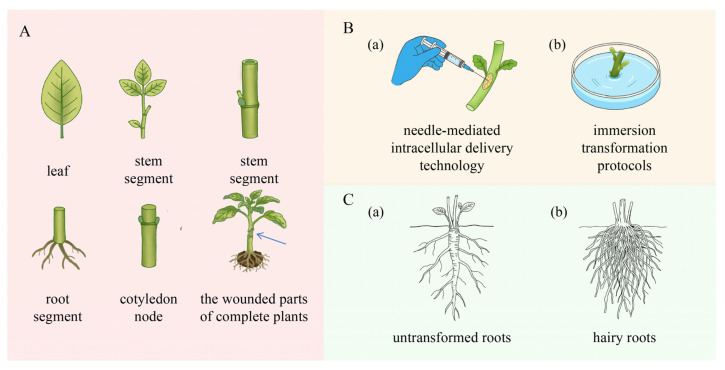
Material selection, methods, and root morphology changes in the genetic transformation of plant hairy roots. (**A**) Illustration of the types of plant tissues that can be used for transformation experiments, including leaves, stem segments, root segments, cotyledon nodes, and wounded parts of the complete plant. (**B**) Schematic showing two commonly used genetic transformation methods: (**a**) needle-mediated intracellular delivery technology and (**b**) the soaking transformation method. (**C**) Comparison of the morphological differences between (**a**) untransformed roots and hairy roots, with (**b**) hairy roots showing greater branching and growth activity.

Explant selection is a key factor determining the efficiency of inducing hairy roots in *R. rhizogenes* and the success of subsequent regeneration. Studies have shown that the type of explant, its physiological age, and genotype can significantly affect the success rate of transformation. The commonly used types of explants include leaves, stem segments, root segments, cotyledon nodes, and the wounded parts of complete plants (Figure 4A). In *C. sinensis* research, leaf and stem segments were used as explants, and formation of adventitious roots was successfully induced through the vacuum osmosis method [66]. In a *Rosa hybrida* study, stem segments and leaves were compared, and it was found that the transformation efficiency of the tender stem segments was higher, reaching up to 74.1% [74]. The physiological state of the explants significantly affects transformation efficiency. Young tissues usually outperform aged tissues. In a study on *Cynanchum stauntonii*, the transformation rate of 20-day-old young stems reached 79.5%, while explants at 60 days old failed to induce formation of adventitious roots [75]. Pre-culturing of embryogenic calluses can enhance transformation efficiency. In a study on *Primula sieboldii*, the hairy-root induction rate after root segments were pre-cultured reached 12% [76]. In recent years, explant selection has tended to be simplified and efficient. In research on *Actinidia valvata*, a high-pressure propagation system was adopted, and formation of adventitious roots was directly induced at the wound sites of living branches, with a transformation efficiency of 50% [77]; in research on *Kalanchoe blossfeldiana*, the leaf-cutting transformation method was used, and transgenic plants were obtained without the need for aseptic operation [78]. In a study on *P. sieboldii*, RUBY markers were successfully utilized to screen out transgenic hairy roots and regenerate complete plants [76]. In conclusion, in explant selection, researchers should comprehensively consider factors such as species characteristics, tissue tenderness, trauma treatment, and visual screening in order to establish an efficient and stable hairy-root genetic transformation system.

The commonly used methods of plant inoculation include needle-mediated intracellular delivery technology and immersion transformation protocols (Figure 4B). For example, an efficient Agrobacterium injection system for *Vigna radiata* seedlings is suitable for gene functional analysis of various plants [79]. In an experimental video published on JoVE, Jedličková et al. (2023) described the process of inducing hairy roots in the inflorescence stems of *A. thaliana* and the hypocotyls of rapeseed via needle injection and demonstrated acquisition of regenerated plants [80]. Wang et al. (2024) reported an efficient infiltration method in which young plant explants were immersed in a bacterial solution of *R. rhizogenes* for infection, successfully resulting in transgenic hairy roots and regenerating plants [81]. In *Frontiers in Plant Science*, Zhou et al. (2022) described the use of the infiltration method to transform hairy roots in combination with CRISPR/Cas9 technology for gene-editing analysis, demonstrating the application potential of this method in functional gene research [49]. The needle-mediated method is suitable for precise injection, and it is often used for injecting a substance into the hypocotyl or stem segments of seedlings. It is easy to employ and suitable for functional gene research. The infiltration method is suitable for large-scale processing, and it is often used for plant leaves, cotyledons, or young plant tissue explants. It is highly efficient and suitable for establishing a stable transformation platform.

In the genetic transformation of hairy roots mediated by *R. rhizogenes*, there are two main categories of screening and identification: analytical detection and morphological observation. Molecular detection is the standard for confirming transgenic events. The most fundamental step is amplifying the reporter gene and the characteristic sequences of the Ri plasmid through PCR. In *P. edulis*, by using eGFP primers and *rolB* gene primers for amplification, researchers verified the successful integration of T-DNA [50]. In *Allium sativum* L., transgenic roots were identified by detecting *rolB* (423 bp) and *rolC* (626 bp), and the presence of Agrobacterium contamination was ruled out by the absence of the *VirG* gene [82]. To further validate transgenic activity, RT-PCR or quantitative real-time PCR (qRT-PCR) is routinely employed to assess the transcriptional expression level of the target gene. The expression of GFP, *rolB*, and *rolC* was detected using RT-PCR in *Citrus medica*, and the stable expression of the *RUBY* gene was confirmed by qRT-PCR in *V. radiata* L. [83,84]. For CRISPR-edited materials, Sanger sequencing is employed to analyze point mutations in target genes. In *S. miltiorrhiza*, the Synthego ICE tool was utilized to quantify editing efficiency and the frequency of homozygous mutations [85]. In addition to molecular detection, morphological characteristics are often used as a preliminary screening basis. The hairy roots induced by *R. rhizogenes* exhibit rapid growth, high branching, dense root hairs, and no gravitropism (Figure 4C). In research on *P. edulis*, hairy roots exhibited a dense, slender, highly branched morphology, with vigorous growth. Meanwhile, untransformed roots presented a state where the main root was clearly visible and the lateral roots grew in an orderly manner [50]. Additionally, in *A. sativum*, it was observed that the hairy roots grew in clusters around the stem disk, showing non-phototropic growth [82]. In addition, hairy roots’ ability to grow autonomously on a culture medium without exogenous hormones is also an important physiological feature, which is directly related to the expression of the *rol* gene in this Ri plasmid.

### 4.2. Establishment of Hairy-Root Regeneration Plant Systems

Callus induction and subsequent plant regeneration constitute fundamental stages in numerous biotechnological applications and genetic improvement protocols [86]. For some endangered or rare species, seeds may encounter difficulties in germination and problems in chromosome preparation, and it may be difficult to observe plant phenotypes and conduct self-pollination studies using hairy roots. Therefore, establishing a complete plant regeneration system can effectively avoid these obstacles. The principles of cell totipotency and pluripotency, as the basis of plant regeneration systems, have been known for decades [87]. However, due to differences in species and tissue types, as well as various obstacles in the regeneration process, achieving whole-plant regeneration remains a major technical challenge [88]. Hairy-root-induced regenerated plants are easy to handle, have high transformation efficiency, and feature well-developed root systems, more segmented roots, and rapid growth. Thus, hairy-root culture offers a feasible method of whole-plant regeneration in plant transformation and has become a widely used plant-tissue-culture technique in plant biotechnology, serving as a stable transgenic plant regeneration system [89]. Efficient regeneration systems have been successfully established for a wide range of horticultural plants, including both herbaceous and woody ornamentals, such as *Ipomoea batatas*, *Ipomoea trichocarpa*, *Panax ginseng*, *Crotalaria juncea*, *L. hybrid*, *P. sieboldii*, and *Brassica species* [41,76].

**Figure 5:**
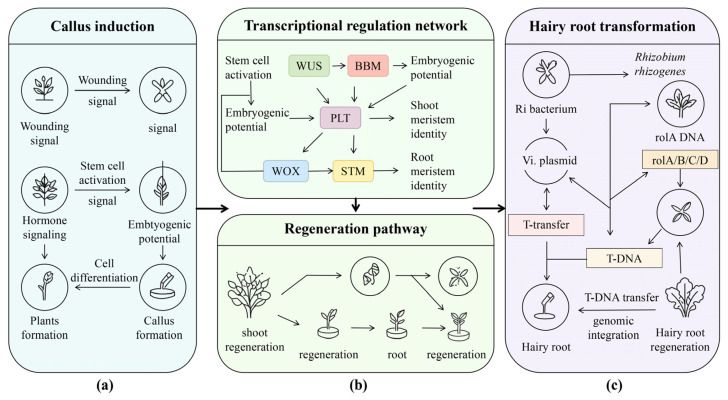
An integrated model of transcription-regulatory networks and hairy-root transformation during plant callus induction and regeneration. This figure systematically synthesizes the interactive relationships between key molecular signaling pathways and morphogenetic programs underlying in vitro plant regeneration. (**a**) Illustration of the synergistic induction of callus formation by wound signals, phytohormone signaling, and stem cell activation cues, followed by plant regeneration via subsequent cellular differentiation. (**b**) The complex regulatory network anchored by core transcription factors such as WUS, BBM, PLT, WOX, and STM, which serve as pivotal hubs in orchestrating stem cell activation, establishment of embryogenic potential, and specification of shoot/root meristem identity. (**c**) Outline of the hairy-root transformation process mediated by *R. rhizogenes*: the *rol* genes (*rolA/B/C/D*) carried on the Ri plasmid are transferred via T-DNA and integrated into the host genome, thereby effectively triggering hairy-root regeneration. The conventional regeneration pathway—whereby a callus undergoes shoot regeneration and root regeneration and ultimately develops into a complete plant—is also depicted.

Following *R. rhizogenes*-mediated transformation, the resulting transgenic hairy roots possess dual regenerative capacity through either callus-mediated organogenesis or direct somatic embryogenesis [90]. The key difference is that callus induction requires hairy roots to produce callus tissue, which is then placed on a selected regeneration solid medium to induce bud formation and regenerate plants (Figure 5). Direct differentiation skips the callus stage, with hairy roots directly inducing seedling formation for continued cultivation [9]. These are the “one-step” and “two-step” methods of plant regeneration.

In the “one-step method,” researchers select excellent hairy roots on a hormone-free MS solid medium for molecular testing and then transfer them to MS medium with different auxin–cytokinin concentrations to induce growth of adventitious buds [91]. The “two-step method” involves cutting excellent monoclonal hairy roots (grown in the dark on a hormone-free MS solid medium) into 2–3 cm segments and transferring them to MS solid medium with different auxin–cytokinin ratios (30 g·L^−1^ of sucrose, pH 5.8) to induce callus-tissue bud formation [92]. After adventitious-bud-growth induction, healthy, non-browning parts of hairy-root callus tissue are selected and transferred to a bud-differentiation medium with different auxin–cytokinin ratios for cultivation [93]. After adventitious-bud-growth induction, plants undergo resistance screening and a rooting culture, gradually forming complete regenerated plants. These are transplanted after testing. Most regenerated plants differ morphologically from ordinary ones, showing wrinkled leaves, shortened internodes, well-developed root systems, and a loss of geotropism [94]. The establishment of the hairy-root system of *Bupleurum chinense* and its regeneration system laid the foundation for the study of secondary metabolites of this species and provided important support for the research and application of genetic transformation mediated by *R. rhizogenes*.

The process by which hairy roots regenerate and develop into a complete plant is not only regulated by exogenous growth-regulatory substances but also dependent on the expression of developmental regulatory genes [95]. In recent years, the crucial roles of transcription factors such as WUS, BBM, and PLT in plant regeneration have been systematically elucidated [96,97,98]. WUS is a key transcription factor that maintains the activity of stem cells in the shoot apical meristem. Its overexpression can directly induce the transformation of somatic cells into the embryonic development pathway [99]. BBM belongs to the AP2/ERF transcription factor family and plays a “molecular switch” role in somatic embryogenesis and the proliferation of embryogenic calluses [100]. In *Z. mays*, co-expression of BBM and WUS2 can bypass the callus formation stage and directly induce the formation of transgenic embryos on embryogenic explants, achieving “one-step” regeneration [100]. The PLT family of transcription factors constitutes the core regulatory factors for the establishment and maintenance of the root stem cell niche. In *A. thaliana*, PLT3, PLT5, and PLT7 control the regeneration from primordia through a two-step mechanism, establish cell pluripotency, and subsequently activate the bud regeneration program [95]. The PLT factors also coordinate redox homeostasis (ROS) by directly activating autophagy-related genes (*ATG8*), providing the necessary cellular environment for the determination of stem cell fate. Through transient or stable overexpression of members of the WUS, BBM, or PLT families, cell totipotency can be induced without relying on exogenous hormones, thereby bypassing the lengthy steps of callus induction and adventitious-bud differentiation in traditional tissue culture processes [96]. In *Malus pumila*, overexpression of *MdWOX5* significantly increased the efficiency with which root primordia transformed into buds [95]. Furthermore, WOUND INDUCED DIFFERENTIATION 1 (WIND1), as a regulatory factor for trauma-induced dedifferentiation, also plays a significant role in promoting the regeneration of shoot organs from explants [100]. These studies indicate that integrating the molecular manipulation of developmental regulatory factors with traditional tissue culture techniques is an important direction for overcoming the bottleneck regarding the efficiency of callus root regeneration and expanding its application scope.

### 4.3. The Process of and Strategies for Optimizing CRISPR/Cas9 Gene Editing in Hairy Roots

CRISPR/Cas-mediated genome editing is a powerful tool in plant functional genomics. Due to the lack of stable transformation and regeneration protocols, as well as the fact that the target shape only appears at the root, hairy-root transformation technology is often used to improve plant shapes. This technology is also used because the transformation mediated by *R. rhizogenes* can rapidly promote regeneration of transgenic biomass, which is crucial for the efficient production of biomedical, pharmaceutical, or industrial molecules [44,101,102]. CRISPR/Cas-mediated genome editing can also be carried out in transgenic hairy roots. A highly efficient genome-editing and hairy-root screening system should consist of three components: a box carrying the gene encoding the CRISPR-related (Cas) nucleases, a box for expressing the guide RNA (gRNA), and a box encoding a selectable or screening marker.

In the CRISPR/Cas9 gene-editing system for hairy roots, the construction of the Cas expression box is the core step in achieving efficient editing. Selection of the correct promoter is the primary consideration. The 35S promoter and its variants are the most commonly used. Ubiquitin promoters (pUbi) from different species such as *A. thaliana*, parsley, and corn are also frequently employed. Tissue-specific promoters can be used to avoid the pleiotropic effects of genes. The *A. thaliana* GATA23 promoter (pAtGATA23) is only active at the earliest stage of lateral root initiation and can be used for tissue-specific knockout. In terms of translation enhancement, the translation enhancer is the 5′ UTR sequence of the *Mac3* gene from japonica rice (*OsMac3*). Studies have confirmed that the 5′ UTR fragment of the *OsMac3* gene (covering the region from the −158 position to 1 bp before the ATG position) has sufficient translation enhancement activity and can be used to improve genome-editing efficiency regarding monocotyledonous and dicotyledonous plants. This *OsMac3* enhancer has been applied in stable transformation systems [103]. In the selection of Cas9 variants, besides SpCas9, which recognizes the classic NGG PAM, researchers have applied Cas9 (VQR), which recognizes NGA PAM and LbCpf1/ttLbCpf1, which recognizes TTTN PAM, etc., in germ cells, expanding the range of targetable editing sites. To ensure that the Cas9 protein effectively enters the cell nucleus and functions, nuclear localization signals (NLSs) need to be fused at both ends of its open reading frame. Usually, two NLSs are sufficient to meet the requirements for nuclear targeting [104,105].

In the CRISPR/Cas9 gene-editing system for hairy roots, the design and testing of gRNA are the key steps that determine editing efficiency and targeting specificity. The gRNA expression cassette consists of a small nucleolar RNA gene promoter, a gRNA sequence, and a transcriptional terminator. The U6 and U3 promoters are recognized by RNA polymerase III to ensure that the gRNA can bind to the Cas protein in the cell nucleus and function properly. In the design of the targeting sequence (crRNA) of gRNA, the length of crRNA is usually 17–20 bp. The target site should be upstream of the NGG PAM sequence, and the GC content should be controlled within 50–70% to enhance the hybridization stability of crRNA with the target DNA. The sequence should avoid poly(T) to prevent RNA polymerase III from prematurely terminating transcription. Additionally, when the pU6 promoter is used, the first nucleotide of the crRNA should be G, and when the pU3 promoter is used, it should be A. For each target gene, it is generally recommended to design at least two crRNAs for screening purposes. After designing is completed, an assessment of gRNA efficiency is conducted, constituting a crucial step to ensure successful editing. The hairy-root transformation system provides an ideal rapid screening platform for this purpose. Hairy-root induction only takes a few weeks, allowing parallel testing of multiple candidate gRNAs within a short period of time [106,107]. In *Phaseolus vulgaris*, a branched root system also enabled rapid assessment of multiple sgRNAs and promoters and identified highly efficient sgRNAs capable of inducing up to 70% frame-shift mutations [108]. In *Solanum tuberosum*, through a branched root culture system, nine candidate gRNAs were rapidly screened, successfully resulting in the efficient editing of the *St16DOX* gene [109]. In terms of bioinformatics prediction tools, platforms such as CRISPR-P v2.0 and CRISPR integrate various plant genome data and can be used to evaluate multiple parameters affecting the efficiency of gRNAs. Integrating a rapid hairy-root transformation system with the gRNA design-evaluation process can significantly shorten the verification period for editing efficiency, providing a reliable technical guarantee for the subsequent screening of efficient gRNAs in stable transformation.

In the CRISPR/Cas9 gene-editing system for hairy roots, the transgenic marker gene is an important tool for distinguishing transformed cells from non-transformed cells. It can be classified into two major categories based on its working principles: selective and screening markers. Selective marking confers growth advantages to transformed cells by applying selection pressure. However, such markers suffer from chimerism: non-transgenic cells escape selection due to protection from adjacent transgenic cells, making it impossible to distinguish between fully transgenic roots and chimeric roots. Screening markers allow visualization and identification of transformed tissues through enzymatic reactions, pigment deposition, or fluorescence signals. Common examples include GUS staining, fluorescent proteins, and pigment synthesis markers. The advantage of these markers is that they can effectively distinguish between fully transgenic and chimeric root tissues.

## 5. Applications of Plant Hairy-Root Genetic Transformation

### 5.1. Research on Improving Plant Molecular Breeding

Plant molecular breeding is a method of cultivating superior new varieties, mainly via plant genetic engineering breeding and molecular-marker-assisted selection [110]. Integration of traditional genetic methods with high-throughput sequencing technology has enabled scientists to decode the genetic basis of desirable traits and uncover the molecular mechanisms underlying them, providing a theoretical foundation for customized crop improvement. In this context, the hairy-root system emerges as a transformative platform, which includes gene function verification, trait enhancement through gene manipulation and RNAi technology, and precision breeding based on CRISPR technology.

The hairy-root system plays a crucial role in verifying the functions of specific genes, enabling rapid intracellular phenotypic analysis of candidate genes. To investigate the function of *PmMYB10.5b* in anthocyanin biosynthesis and the purple pigmentation of *Prunus mume*, researchers established an efficient hairy-root genetic transformation system. Transgenic hairy roots overexpressing *PmMYB10.5b* exhibited pronounced red pigmentation in both callus and root tissues, accompanied by a marked elevation in anthocyanin levels, thereby producing a distinct red phenotype [77].

In regard to trait improvement, hairy roots are utilized in studies on functional gain and loss to enhance stress resistance and other agronomic traits. Hairy-root-mediated plant genetic transformation overcomes inter-specific isolation, improves varieties, and leads to innovations in germplasms in a manner superior to that of traditional breeding [48]. By utilizing *R. rhizogenes*, exogenous genes can be introduced and integrated into the plant genome, leading to the expression of desirable traits and the enhancement of plant varieties. Transgenic *G. max* roots overexpressing *GsEXLB14* mediated by rhizobia showed significant increases in relative growth values in terms of quantity, length, and weight under salt and drought stress conditions, and the activity of oxidase was also significantly enhanced [111]. Besides gene-expression introduction, some plants boost hairy-root stress resistance via gene silencing, such as how silencing *AhHDA1* in *Arachis hypogaea* improves drought resistance [112].

RNAi is an effective method that induces degradation of homologous mRNA through double-stranded RNA, thereby achieving post-transcriptional gene silencing. By combining RNAi technology with the hairy-root transformation system, it provides a powerful tool for rapid and efficient research on plant gene functions [113]. Using Agrobacterium-mediated hairy-root transformation, RNAi silencing of the *GUS* gene was achieved in *Lotus japonicus* by expressing hpRNA. More than 60% of the hairy roots showed a significant reduction in or complete silencing of *GUS* activity. *GUS* gene silencing was also observed in the symbiotic root nodules at both the early and late stages of nodule organogenesis. This study demonstrates that the hairy-root RNAi system is a powerful tool for investigating gene functions in roots and root nodules [114]. In *Ipomoea trifida* hairy-root composite plants, when the *SlGRAS18* and *SlGRAS38* genes were silenced through RNAi, researchers observed that the mycorrhizal infection was delayed, while *SlGRAS43*-RNAi roots showed an increase in mycorrhizal colonization [115]. Compared with traditional construction of stable transgenic plants, the adventitious-root RNAi system can yield transgenic root systems within several weeks and evaluate the gene-silencing effect. It is particularly suitable for species that are difficult to stably transform using *A. tumefaciens* [116]. Moreover, the single-cell nature and absence of chimeras of the adventitious roots ensure the reliability of the gene-silencing phenotype.

In CRISPR-mediated precision-breeding technology, the hairy-root system is utilized as a rapid screening platform for gene editing, and it is particularly suitable for woody and recalcitrant species. In *C. sativus*, a system based on adventitious roots can simultaneously assess transgene expression and CRISPR/Cas9 editing activity within one month after transformation, providing a valuable tool for functional research on this difficult-to-transform species [60]. In *Lactuca sativa*, an adventitious root CRISPR platform targeting the shade avoidance response gene *LsHB2* was optimized by combining plant-derived promoters with the At-pU6-26 variant, significantly enhancing editing efficiency and enabling rapid genotyping and insertion–deletion mutation analysis [117]. Moreover, this method has also been extended to woody and difficult-to-transform species. In *Vitis vinifera*, adventitious-root induction was combined with the siRNA tagging system to evaluate the activity of the adenine base editor (ABE8e), achieving targeted A-to-G conversion and promoting lignin deposition by knocking out the *MYB4a* gene [118]. In *Prunus amygdalus*, a composite plant consisting of wild-type stem segments and transgenic adventitious roots was constructed, and the efficiency of the guide RNA was evaluated by knocking out key transcription factor genes (*ETHYLENE RESPONSE FACTOR 74* and *GIBBERELLIC ACID INSENSITIVE*) [119]. In *G. hirsutum*, the optimized adventitious root system served as a reliable validation platform, allowing sgRNA screening and editing efficiency assessment within days to weeks, thereby facilitating the determination of the optimal target site for stable transformation [120]. These examples demonstrate that adventitious-root-mediated gene editing not only accelerates the initial screening of editing reagents but also provides an efficient and time-saving alternative for species that are difficult to transform or have long generation cycles, significantly advancing the development of molecular breeding processes.

Apart from brief assessment, transgenic hairy roots can also achieve stable plant regeneration through hormone supplementation, thereby yielding genetically stable offspring. In research on *Gossypium arboreum* L., using the cotyledon explants of variety No. 24, researchers established a transformation protocol based on perforation-assisted inoculation, achieving a rate of sterile hairy roots equal to 82.6%. Overall, 82% of the hairy roots produced embryogenic calluses, and, for the first time, stable transformed *G. arboreum* L. plants were regenerated through somatic embryogenesis after 7 months of culture, providing a useful and reliable platform for gene function analysis regarding *G. arboreum* L. [63]. This method also achieved a high conversion efficiency in *S. miltiorrhiza*. The existence of transgenic lines was confirmed by PCR testing [61]. Transgenic-hairy-root-derived regenerated plants often have heritable variant traits like dwarfism, showing their potential in garden-plant breeding. Additionally, these plants typically display well-developed root systems and vigorous fibrous root growth, further underscoring the utility of hairy-root-mediated transformation in plant improvement.

Despite these significant advancements, there are still some challenges in using the hairy-root system for molecular breeding, including reliance on transformation methods specific to certain genotypes, the instability of transgene expression during long-term subculture processes, and the difficulty of expanding the applicability of regeneration methods for many woody plants.

### 5.2. Sustainable Production of Secondary Metabolites via the Genetic Transformation System for Plant Hairy Roots

Plant secondary metabolites are a large class of organic compounds in plants that are not essential for plant growth and development [121]. Their production relates to plant species, organs, tissues, and growth stages. They are crucial for plant adaptability and symbiotic relationships and constitute important resources in modern society. Plant secondary metabolites serve as raw materials for drugs and food additives with economic value in medicine and industry [122]. These metabolites are synthesized in plants naturally or created via tissue/cell cultures, but plant-inherent susceptibility to pests, diseases, and the environment leads to low and unstable production, falling short of market demand [123]. Hairy roots, highly differentiated tissue cultures with fully expressed metabolic pathways, offer genetic and biochemical stability, rapid growth on hormone-free media, lower costs, and a simple way to synthesize secondary metabolites [124]. Thus, hairy-root culture is widely used. Phytochemical analyses of hairy-root cultures reveal the production of multiple bioactive compound classes, including nitrogen-containing alkaloids, glycosylated secondary metabolites, flavonoid derivatives, quinoid compounds, and structural polysaccharides [125].

Hairy roots have been successfully utilized for the production of various active compounds used in medicine. A *Daucus carota* L. hairy-root culture method for anthocyanin production was established, serving as an in vitro approach to generating both anthocyanins and antioxidants while providing novel insights for studying secondary-metabolite regulation in carrots [126]. MeJA stimulated a significant increase in plumbagin in the hairy roots of *Plumbago auriculata*, which has great potential as an inhibitor of many human and agricultural pathogens, providing exciting research ideas for expanding the development of biopesticide inhibitors [127]. Also, natural-product drug research has shown that secondary metabolites benefit human health, hence *Verbascum thapsus*’s anti-inflammatory use due to its bioactive molecules [128]. Nowadays, hairy roots are widely used for large-scale production of various important drug compounds, such as paclitaxel, ginsenosides, vincristine, vincristine sulfate; pigment substances such as shikonin and betaine; and special chemicals such as indigo. In *S. miltiorrhiza*, hairy roots were used as a heterologous expression platform. By introducing the taxane synthase gene (*TwTS*) from Taxus and knocking out the competing pathway *SmCPS1* as well as overexpressing *SmWRKY61*, researchers increased the yield of taxane to 65.17 ± 5.25 mg/kg fresh weight [129]. In *Panax quinquefolius*, when hairy roots were treated with linalool as a new inducer, 0.1 µM linalool treatment for 24 h increased the total saponin content by 29% [130]. These studies demonstrate that by combining genetic engineering with induced regulatory strategies, the hairy-root system has great potential for use in the efficient production of drug compound components. Furthermore, the medicinal active ingredients are also applicable in the formulation of skin care products. The cosmetics industry is increasingly focusing on plant-based bioactive compounds with anti-aging, whitening, and antioxidant properties. Hairy-root culture provides a sustainable, pollution-free, and scalable source of these high-value cosmetic active ingredients. Compared with traditional plant extraction, hairy-root culture offers a controlled and year-round producible system.

In addition to their use for creating drug compounds, hairy roots have been used to produce a natural pigment and as an important platform for the sustainable production of specialty chemicals. The sugar beet hairy-root culture system has been systematically optimized for the efficient production of the natural pigment betacyanin. By systematically screening various biological and non-biological inducers and establishing the “late exponential phase addition” strategy, researchers significantly increased the yield of betacyanin. This strategy was successfully scaled up and verified in a bubble column bioreactor, confirming the feasibility of using hairy-root culture as a platform for the industrial production of natural pigments [131]. Metabolic engineering research on pigment biosynthesis in hairy roots has attracted a great deal of attention. Moreover, the genetic stability and scalability of hairy-root cultivation make it particularly attractive for industrial applications in the textile, food, and cosmetic industries, where there is an increasing demand for natural and sustainable sources of colorants.

Hairy-root culture systems not only offer advantages in the production of plant secondary metabolites but have also become an important platform in molecular agriculture—that is, using plant systems to produce recombinant proteins—in recent years [58]. Compared with traditional plant cell suspension culture, hairy roots offer significant advantages, such as high genetic stability, rapid growth in hormone-free media, simple downstream processing, and the ability to be scaled up in bioreactors. Moreover, hairy roots can secrete recombinant proteins into culture media, not only improving the homogeneity of the product but also significantly simplifying the purification process and reducing production costs [13,132]. In recent years, significant progress has been made in the expression of medicinal proteins: recombinant IDUA was successfully secreted and expressed in the hairy roots of *Brassica rapa*, which is a biologic agent of great medical value; in the filiform roots of *Nicotiana tabacum*, a medicinal protein, ocriplasmin, used for treating vitreous macular adhesion, was successfully expressed [133]. Overall, filamentous roots serve as a recombinant protein expression platform, possessing advantages such as low production costs, no risk of human pathogens, eukaryotic post-translational modification capabilities, and bioreactor scalability.

The hairy-root culture system is a versatile and robust platform that can be used for the sustainable production of a variety of high-value compounds ranging from medicinal components to natural pigments. The genetic stability of hairy roots makes them highly adaptable to metabolic engineering, in turn rendering them a powerful alternative to field cultivation and microbial production systems. However, the commercialization of these applications is still hindered by several major challenges, including the limitations of bioreactors, inconsistent responses to inducers, and long-term genetic instability.

### 5.3. Application of Plant Hairy Roots for Environmental Remediation

As pollution from human settlement increases, factors like pollution’s persistence and large scale make existing physical and chemical environmental remediation technologies costly and ineffective. Thus, an optimized, efficient pollutant-absorbing, -metabolizing, and -tolerating control technology is urgently needed. In recent years, research on hairy roots has shown that they offer advantages in phytoremediation, being efficient, safe, easy to use, and eco-friendly. As roots are the main plant organs in contact with pollutants and physiologically similar to real roots, they can degrade harmful compounds via unique metabolic pathways, becoming a fast and efficient tool for environmental remediation [134]. Hairy-root tissue is a great model for identifying phytoremediation limits, understanding toxic-compound metabolism, and analyzing plant–microorganism interactions in pollution removal. It is suitable for studying heavy-metal absorption, pollutant-related proteins, toxicity, and plant stress responses [135].

Hairy roots were first applied in the field of plant remediation for the removal of inorganic pollutants from soil. Studies have shown that hairy roots can accumulate heavy metals within themselves without presenting significant metal toxicity or causing cellular damage. Boominathan R and Doran PM studied heavy-metal absorption in *Thlaspi caerulescens* and *Alyssum bertolonii* hairy roots [136]. Hairy roots of super-accumulative plants are used to study radionuclide extraction and accumulation in roots. *Brassica juncea* and *Chenopodium amaranticolor* hairy roots can remove up to 5000 μM of uranium from sewage within a short incubation period [137]. In addition, hairy roots can also be used to screen the tolerance, accumulation capacity, and removal efficiency of different plant varieties with respect to pollutants such as polychlorinated biphenyls, heavy metals, and radioactive nuclides, providing a theoretical basis for the breeding of plant remediation varieties.

There has been great progress in using hairy roots to remove organic and inorganic environmental pollutants. First used in phytoremediation for soil inorganic-pollutant removal, hairy roots can accumulate pollutants without producing metal toxicity or causing cell damage. Organic pollutants like the phenolic ones from domestic sewage and petrochemical sources, which enter the environment through pesticide use and aromatic-pollutant degradation, threaten human health. In recent years, studies have shown that hairy roots of *Sinapis alba*, *Solanum lycopersicum*, and *D. carota* can remove phenols and 2,4-DCP. Screening and mass-producing enzymes from hairy roots can allow large-scale phenolic-pollution treatment. The hairy roots of *Solanum aviculare* Forst are very efficient in remediating these pollutants [138]. Additionally, hairy roots have led to breakthroughs in medical pollutant-elimination research, with *Helianthus annuus* hairy roots and exudates having been used to degrade tetracycline and oxytetracycline in wastewater [139].

Dyes are among the main pollutants in textile industrial wastewater. These synthetic fuels have complex aromatic ring structures, high stability, and strong toxicity. Traditional treatment methods cannot completely degrade them, and they are also costly. Hairy roots possess a highly branched root structure and an abundance of oxidoreductase enzymes, demonstrating great potential for application in dye degradation and wastewater decolorization. In recent years, there has been significant progress in research on the use of hairy roots in the biological remediation of textile dyes. The hairy roots of *Brassica napus* can tolerate up to 480 µg/mL of Naphthol blue-black. At a concentration of 180 µg/mL of Naphthol blue-black, the removal rate can reach 100% within 10 days. Meanwhile, the biomass of the hairy roots can be reused for three decolorization cycles, maintaining a removal rate of 55–60%. The removal mechanism mainly involves passive adsorption in the initial stage, and, in the later stage, it initiates active metabolism by regulating the expression of proteins such as hydrolases and redox enzymes [140]. The *R. rhizogenes* strain NCIM 5140 was used to infect leaf explants of *Sesuvium portulacastrum*. The induction rate for hairy roots reached 70%. The degradation rate of active green 19A-HE4BD by the hairy roots was 98%. The degradation of the dye was confirmed by HPLC and FTIR analysis, and a seed germination test proved that the degradation metabolites were non-toxic. This study is the first report on inducing formation of hairy roots in *S. portulacastrum* and using them for plant-based textile dye remediation [141]. Compared with traditional physical–chemical methods, dye degradation mediated by hairy roots has the advantages of low operating costs, environmental friendliness, and reusability, and it is particularly suitable for the pretreatment or comprehensive treatment of wastewater from concentrated areas in the textile industry.

Hairy roots have shown excellent application prospects in three fields of environmental remediation: heavy metals, organic pollutants, and industrial dyes. As a safe, efficient, and scalable green remediation tool, they are attracting increasing attention.

## 6. Discussion

The hairy-root genetic transformation system holds broad application prospects in areas such as gene function characterization, molecular-breeding improvement, secondary-metabolite production, and environmental remediation. At the molecular level, the mechanism of Ri plasmid T-DNA transfer and integration mediated by *R. rhizogenes* provides a theoretical foundation for introducing foreign genes. At the technical level, combination of CRISPR/Cas9 gene editing with hairy-root transformation offers an efficient platform for gene function research and precision breeding. At the application level, the hairy-root system has expanded from single-purpose secondary-metabolite production to diverse fields including molecular-breeding enhancement and environmental bioremediation. However, research on hairy roots, from laboratory studies to practical applications, is still hindered by numerous challenges.

Genetic transformation of hairy-root plants can boost crop yields and stress resistance by introducing desired traits. Inserting specific genes improves crop traits and tolerance to biotic and abiotic stress. Although the hairy-root genetic transformation system is being optimized, many plant species and crop genotypes are unsuitable for tissue culture or have low transformation efficiency, making effective gene transformation a challenge for many crops [94]. The hairy-root genetic transformation system has been successfully applied to various dicotyledonous plants, but slow progress has been made in monocotyledonous plants. Although root-inducing Agrobacterium-mediated gene transformation has achieved a certain degree of success in crops for which genetic manipulation is relatively convenient such as *Z. mays*, the induction efficiency of hairy roots in monocotyledonous plants is generally low, which restricts the widespread application of this technology to major food crops like *Oryza sativa* and *Triticum aestivum* [142]. Hairy-root-formation-induction efficiency is influenced by multiple factors, such as strain type, explant material, infection conditions, and culture conditions, in a synergistic manner. By optimizing vector construction, explant type, strain selection, and co-culture system, along with using highly toxic root-inducing *R. rhizogenes* strains and suitable marker genes, researchers can improve the transformation efficiency of monocotyledonous plants, expanding the applicational scope of hairy-root technology. The transformation efficiency of hairy roots varies significantly not only among species but also among different genotypes within the same species, making them difficult to apply to a wide range of genetic resources. The seeds of hairy-root plants are largely difficult to germinate and not resistant to dehydration or low temperatures. Additionally, conventional low-temperature storage techniques cannot achieve effective preservation. This further increases the difficulty of constructing transformation systems. In terms of recombinant protein expression, the hairy-root system is hindered by problems such as low expression levels, instability, and complex gene expression regulation, which affect stable production and the reliability of gene function research results. Moreover, industrial-scale amplification of hairy roots is hindered by severe mass-transfer limitations. Hairy roots’ dense network structure is likely to form oxygen and nutrient gradients in liquid media, while traditional stirred reactors can cause mechanical damage. Currently, there are no reactor designs that can achieve an ideal balance between low shear force and high mass-transfer efficiency. In terms of biosafety, the release of transgenic hairy roots into the environment may pose risks such as horizontal gene transfer and changes in microbial community structure. These limitations indicate that this system still needs further optimization and systematic evaluation through practical applications. Also, transgenic-hairy-root biosafety requires further study, despite the short history of hairy-root culture and the Ri plasmid’s advantages as a genetic-engineering vector [143]. In the future, when transgenic hairy-root bud regeneration technology is more optimal, it may become a better gene-transfer system than the Ti plasmid [99]. For woody plants and difficult-to-transform species, the combination of the hairy-root transformation system and CRISPR/Cas9 gene-editing technology has achieved significant breakthroughs: the first CRISPR editing of hairy roots was achieved in *V. vinifera*, the efficiency of gRNA was rapidly evaluated in *P. amygdalus* through composite plants (wild-type above-ground parts and transgenic hairy roots) [144,145], and gene-editing systems mediated by hairy roots were also established in *A. chinensis* and *C. sinensis* [66,71]. These advancements indicate that the hairy-root system is becoming an effective tool with which to overcome the genetic transformation bottleneck relating to woody plants.

Although cultivation of hairy roots has led to many breakthroughs on a laboratory scale, it is still hindered by numerous challenges in commercial applications. Filamentous roots are types of adherent tissue. Their dense network structure is prone to forming “anoxic zones” in liquid culture media, resulting in low efficiency of oxygen and nutrient conduction. Therefore, the lack of suitable bioreactors is the primary obstacle hindering the commercial application of adventitious roots. Furthermore, in processes wherein hairy roots are involved in regulating metabolic pathways, although overexpression of a single key gene can increase the yield of the target product, the dynamic regulatory mechanism of the entire metabolic network remains unclear. Hairy roots exhibit high genetic stability. However, during long-term continuous subculture, phenotypic drift still occurs, resulting in a gradual decline in the productivity of high-yield clones [146]. DNA methylation, transposon activation, or chromosome aberrations may occur, seriously hindering the long-term maintenance and standardized production of high-yield strains. However, the molecular mechanisms underlying these phenomena remain unclear [147,148].

The molecular mechanism of Ri plasmid T-DNA transfer and integration in *R. rhizogenes* roots has provided a theoretical foundation for genetic transformation. The optimization of cultivation conditions and the establishment of the CRISPR/Cas9 editing process have offered technical support for efficient gene manipulation. The expansion in the application of hairy-root systems in three major fields—molecular breeding improvement, secondary-metabolite production, and environmental bioremediation—constitutes a complete “mechanism–technology–application” innovation chain. However, the transition of hairy-root technology from laboratory research to practical applications is still hindered by multiple bottlenecks, such as species-dependent transformation efficiency, limitations in mass transfer during large-scale cultivation and when reactor design flaws arise, the “ceiling effect” of metabolic regulation, the instability of inducer effects, and phenotypic drift during long-term cultivation. Only when hairy-root technology achieves a coordinated breakthrough in these several aspects can it truly successfully leap from the laboratory to industry, thus providing strong technical support for plant biotechnology, sustainable agriculture, and green biomanufacturing.

## Figures and Tables

**Table 1:** Recent studies on genetic transformation systems for hairy roots in plants. “-” indicates data not reported in the original publication. Efficiency values are as reported by each study.

Plant Species	Most Responsive Explant Type	Bacterial Strain	Transformation Trend	Efficiency	Factors Associated with Efficiency > 70%
*Cucumis sativus* L. [60]	Cotyledon	K599	Herbaceous	100%	1. Strain chosen: K599. 2. Co-culture duration: 5 days is the optimal duration; shorter or longer durations will reduce efficiency. 3. Culture solution concentration: OD_600_ = 0.4.
*Salvia miltiorrhiza* [61]	Leaf	Ar.qual	Herbaceous	73.85%	1. Bacterial solution concentration: OD_600_ = 0.6. 2. Inoculation time: 10 min. 3. Co-culture time: 3 days. 4. Screening agent concentration: 7.5 mg/L of ampicillin effectively inhibits non-transformed explants.
*G. max* [62]	Hypocotyl	K599	Herbaceous	-	-
*G. hirsutum* [63]	Cotyledon	AR1193	Herbaceous	12%	-
*Litchi chinensis* [64]	Stem segment	MSU440	Woody	9.33% (overall conversion efficiency)	-
*Fragaria vesca* [65]	Hypocotyl	MSU440/C58C1	Herbaceous	71.43% (Positive eGFP root frequency)	1. Strain selection: MSU440 or C58C1 is superior to Ar1193 and K599. 2. Tissue culture material: The lower hypocotyl provides the best result. 3. Optimized parameters: OD_600_ = 0.7, infiltration for 10 min, co-culture for 4 days.
*Camellia sinensis* [66]	Leaf	ATCC15834	Woody	Maximum 14.23%	-
*Solanum erianthum* [67]	Leaf	A4	Herbaceous	72%	1. Bacterial solution concentration: OD_600_ = 0.6–0.8. 2. Co-culture time: 2 days. 3. Dark culture: Induction under complete darkness and inhibition by light. 4. There is no need for acetylsalicylic alcohol (AS)
*Typha domingensis* [68]	Somatic embryo	K599	Herbaceous	68%	-
*Matricaria chamomilla* [69]	Rootless plant wound	ATCC15834	Herbaceous	-	-
*Trigonella foenum-graecum* [70]	Leaf	MSU and 15834	Herbaceous	-	-
*Actinidia chinensis* and *Actinidia eriantha* [71]	The leaves, petioles, and stem segments of young tissue from culture plants	K599	Woody	80%	1. Unlabeled transformation: There is no need for antibiotic screening, simplifying the process. 2. Infection time: 10~13 min is the optimal duration. 3. Root tip removal method: Removing the tip of the root can increase regeneration efficiency from 10% to 37%. 4. Applicable to multiple varieties: Effective for both ‘Hongyang’ and ‘White’.
*Hyoscyamus muticus* [72]	Hypocotyl	A4	Herbaceous	73.4%	1. Strain selection: Strain A4 is significantly superior to strain 15834. 2. Callus: The hypocotyl shows the strongest response. 3. Silver nanoparticle induction: 100 mg/L of AgNPs results in peak levels of total alkaloids, scopolamine, and hyoscyamine.
*Verbascum erianthum* and *Verbascum stachyiforme* [73]	Leaves at 21 days of age	A13	Herbaceous	80.55%	1. Strain selection: A13 belongs to the Mitchi strain, containing 12 T-DNA transfer enhancer sequences, with strong virulence. 2. Age of explants: The best age is 21 days, and the effect significantly decreases by 14 and 28 days. 3. Inoculation method: Direct injection into 3 wounds is superior to 5 wounds. 4. Co-culture time: 72 h is better than 48 h. 5. Optimization of culture medium: B5 + 1.5 mg/L of NAA can inhibit browning, and the growth index can reach 20.42.

## Data Availability

This research did not involve generation of new primary datasets. All relevant data cited in the manuscript are available from the corresponding author upon reasonable request or via the referenced datasets.
